# Choroidal Vascularity Features in Patients with Choroideremia and Cystoid Spaces

**DOI:** 10.3390/diagnostics11030382

**Published:** 2021-02-24

**Authors:** Claudio Iovino, Valentina Di Iorio, Francesco Testa, Viviana Bombace, Paolo Melillo, Kiran Kumar Vupparaboina, Jay Chhablani, Francesca Simonelli

**Affiliations:** 1 Eye Clinic, Multidisciplinary Department of Medical, Surgical and Dental Sciences, University of Campania ‘Luigi Vanvitelli’, Via Pansini 5, 80131 Naples, Italy; claudio.iovino1@unicampania.it (C.I.); valentina.diiorio@unicampania.it (V.D.I.); francesco.testa@unicampania.it (F.T.); viviana.bombace@gmail.com (V.B.); paolo.melillo@unicampania.it (P.M.); 2 Smt. Kanuri Santhamma Centre for Vitreo-Retinal Diseases, L V Prasad Eye Institute, Hyderabad 500034, India; Kiran1559@gmail.com; 3 Department of Ophthalmology, University of Pittsburgh Medical Center, Pittsburgh, PA 15213, USA; jay.chhablani@gmail.com

**Keywords:** choroidal vascularity index, choroideremia, cystoid macular edema, cystoid space

## Abstract

Cystoid spaces (CSs) are a common retinal finding in choroideremia (CHM) patients. The aim of this study was to analyze the vascular features of the choroid associated with the presence of CSs in patients with confirmed genetic diagnosis of CHM. A total of 33 patients (33 eyes) were enrolled in this retrospective cross-sectional study and divided into two groups based on the presence (17 eyes) or absence (16 eyes) of CSs. Choroidal features were evaluated on spectral-domain optical coherence tomography including subfoveal choroidal thickness (CT), total choroidal area (TCA), luminal choroidal area (LCA), and stromal choroidal area (SCA). The choroidal vascularity index (CVI) was then calculated in all study eyes. All structural choroidal parameters were calculated both on the entire length of the B-scan and in the central subfoveal 1500 μm. The average age was 37.3 ± 11.6 and 31.4 ± 16.7 years (*p* = 0.25) and mean logMAR best-corrected visual acuity was 0.11 ± 0.20 and 0.20 ± 0.57 (*p* = 0.54) in the CHM groups with and without CSs, respectively. There were no significant differences in subfoveal CT, and TCA, LCA, SCA, and CVI evaluated on either the entire scan or in the central 1500 μm (all *p* > 0.05). All choroidal vasculature parameters exhibited no significant differences between CHM eyes with and without CSs. Our results suggest that the choroid may not be involved in the development of CSs in patients with CHM.

## 1. Introduction

Choroideremia (CHM) is an X-linked chorioretinal dystrophy characterized by a diffuse, progressive atrophy of the choroid, retinal pigment epithelium (RPE), and retina [1]. The exact pathogenesis of the disease has not been completely elucidated. It is unclear whether the initial events primarily begin in the RPE progressing into the degeneration of the choroid and photoreceptors, or the photoreceptors are involved first, followed by RPE and choroid impairment [1,2].

CHM is remarkable for chronic progressive visual loss, including early onset night blindness and peripheral visual field reduction [3,4]. Patients usually retain good central visual acuity into the fifth decade of life, despite structural alterations of the central retina in the early stage of the disease [5,6].

Cystoid spaces (CSs) are a common retinal finding in CHM patients, with a prevalence of up to 63% as detected by spectral-domain optical coherence tomography (SD-OCT) [7]. The pathogenesis of CSs in eyes with CHM is not well understood, and different mechanisms have been proposed including Muller cell degeneration, blood–retinal barrier disruption, and RPE dysfunction [8]. To date, there has been limited investigation of the choroid in relation to the presence of CSs in patients with CHM. The choroidal vascularity index (CVI) provides the capability to calculate quantitative parameters of the choroid and stratify the stromal and vascular components [9,10].

Recently, Murro et al. reported no differences in the CVI between young CHM patients and controls [11]. Nevertheless, they did not make any distinctions between eyes with or without intraretinal cysts. Conversely, the choroid was proposed to be an important factor to consider in the etiology of cystoid macular edema (CME) in patients with retinitis pigmentosa (RP) [12].

The aim of this study was to examine in detail the vascular features of the choroid associated with the presence of intraretinal CSs in patients with confirmed genetic diagnosis of CHM.

## 2. Methods

In this cross-sectional study, patients with CHM were retrospectively evaluated at the Referral Center of Hereditary Retinal Dystrophies of the University of Campania “Luigi Vanvitelli.” Institutional review board approval was obtained for a retrospective consecutive chart review. The study adhered to the guidelines of the Health Insurance Portability and Accountability Act and was performed in accordance with the tenets of the Declaration of Helsinki.

### 2.1. Patients and Clinical Examination

Patients with a confirmed genetic diagnosis of CHM were reviewed and divided into two groups based on the presence or absence of CSs.

Clinical diagnosis of CHM was based on the patient’s history of nyctalopia and/or hemeralopia, presence of the characteristic fundus findings (including choroidal and RPE degenerative changes throughout the posterior pole and mid-peripheral retina), variable degree of peripheral field restriction, and electroretinogram (ERG) abnormalities. In all patients, DNA and total RNA were extracted from peripheral blood leukocytes and exons 1 to 15 of the *CHM* gene were amplified by PCR as previously described [13].

CSs were defined as one or more fluid-filled, intraretinal cysts in the macula as detected by volumetric SD-OCT. In patients with bilateral CSs, the eye with greater central macular thickness (CMT) was selected for analysis.

In CHM patients without CSs, the eye with the best quality image was selected for the analysis.

Exclusion criteria included the presence of any other retinal disease or ocular disorder that could affect choroidal thickness (CT) or could be associated with the development of CSs (e.g., recent history of cataract and/or vitreoretinal surgery in the previous 6 months), hyperopia, or myopia > 6 diopters. Additionally, patients with media opacities that could influence image quality were also excluded from the study.

For eligible patients, deidentified medical records and multimodal imaging findings were comprehensively reviewed. All patients underwent a complete ophthalmological examination encompassing the Snellen best-corrected visual acuity (BCVA) with logMAR conversion for statistical analysis, slit-lamp biomicroscopy of anterior segment and fundus examination, intraocular pressure, Goldman visual field (GVF), full-field ERG by corneal contact lens electrodes with a Ganzfeld stimulator (Roland Consult, Brandenburg an der Havel, Germany), and SD-OCT and fundus autofluorescence (Heidelberg Spectralis HRA + OCT; Heidelberg Engineering, Germany). Microperimetry was performed by an automatic fundus-related perimeter (MP1 Microperimeter, Nidek Technologies, Padova, Italy). History of any medication for the intraretinal fluid was also investigated, considering the potential influence of some drugs (anti-vascular endothelial growth factors, carbonic anhydrase inhibitors, etc.) on the choroidal vasculature.

### 2.2. OCT Analysis of the Retina and Choroid

A 20° × 20° volume scan was obtained for all study eyes and the horizontal 6 mm B-scan section through the central fovea was used for the analysis, as it is representative of the total macular choroidal vascularity [14]. CMT was automatically displayed by the thickness profile module.

Subfoveal CT was manually measured on the horizontal foveal OCT B scan using the caliper tool of the built-in automated software as the distance between Bruch’s membrane interface and the sclerochoroidal interface.

The exact location of the CSs in the retinal layers was recorded using horizontal linear OCT B-scans. The ellipsoid zone (EZ) and external limiting membrane (ELM) integrity were evaluated in the foveal 1500 μm and in the area under the intraretinal fluid. The presence of outer retina tubulations (ORTs) was also ascertained.

**Figure 1:**
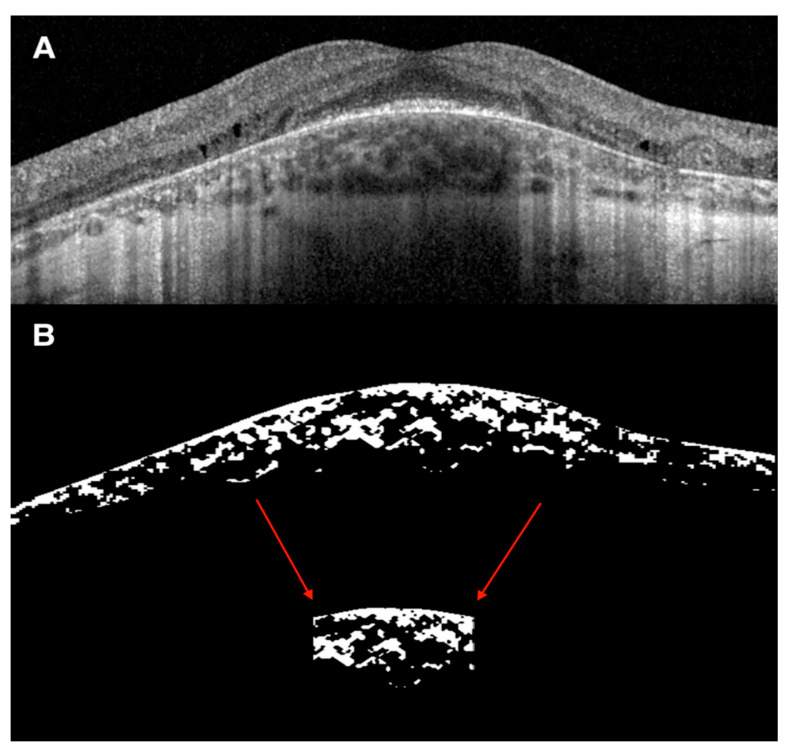
Representative case of choroidal vascularity index (CVI) evaluation on spectral-domain optical coherence tomography (SD-OCT) image. (**A**) SD-OCT 20° or 6 mm B scan of a 21-year-old man with CHM and intraretinal cysts in the inner nuclear layer with underlying disruption of the outer retinal layers. (**B**) Binarized SD-OCT 20° or 6 mm B scan image showing the total choroidal area (TCA) measured as the sum of the stromal choroidal area (bright regions) and the luminal choroidal area or LCA (dark regions). The CVI was calculated as the ratio of LCA over TCA. The same choroidal parameters were measured on the subfoveal 1500 μm (red arrows).

The CVI was calculated using the previously reported automated algorithm that included initial denoising with localization of the choroidal inner and outer boundaries [15,16,17]. To allow computation of the luminal choroidal area (LCA) and stromal choroidal area (SCA), the OCT B-scan passing through the fovea was binarized and choroidal components were segmented. The bright regions were labelled as SCA and the dark regions as LCA. Total choroidal area (TCA) was measured as the sum of the SCA and LCA, and the CVI was calculated as the ratio of LCA over TCA. All choroidal parameters were calculated both on the entire length of the B-scan (6 mm) and in the subfoveal 1500 μm (Figure 1).

### 2.3. Statistical Analysis

Continuous variables are reported as mean ± standard deviation (SD) and categorical variables are reported as counts (percentage). *T*-tests and Fisher’s exact tests were adopted to explore differences in the study parameters between the patients with and without CSs for continuous and categorical variables, respectively. In order to correct for multiple comparisons, *p*-values were compared with Benjamini–Hochberg’s (BH) adjusted α with a false discovery rate of 5%.

## 3. Results

A total of 33 patients (33 eyes) from 26 families met the inclusion criteria and were divided into two groups based on the presence (17 eyes) or absence (16 eyes) of CSs. The pathogenic mutations of the *CHM* gene are shown in Table 1.

All patients’ demographics and retinal and choroidal characteristics of the study participants are summarized in Table 2.

Neither group exhibited any significant differences as pertaining to age and BCVA. In the CS group, intraretinal cysts were unilateral in 10 eyes (58.8%) and bilateral in 7 eyes (41.2%). None of the patients with intraretinal cysts were taking any medication.

### OCT Analysis of the Retina and Choroid

In the CS group, cysts were located exclusively in the inner nuclear layer (INL). Analysis of the outer retinal bands in the subfoveal 1500 μm revealed no statistical differences as for EZ and ELM integrity (all *p* > 0.05, Table 2).

Moreover, analysis of the outer retinal bands under the cysts revealed that ELM and EZ were intact only in 2 eyes (11.8%).

As expected, the mean CMT was greater in the CS group with intraretinal cysts (272.3 ± 57.7 vs. 232.1 ± 69.3), albeit the difference was not statistically significant (*p* = 0.08, Table 2). Conversely, mean subfoveal CT was greater in the CHM group without versus with CSs but still not significant (*p* = 0.26, Table 2). ORTs were present in 17 eyes (100%) in the CS group and 11 eyes (68.7%) in the group without cysts, and the difference was statistically significant (*p* = 0.02).

All data for choroidal vasculature analysis are summarized in Table 3.

TCA, LCA, and SCA were all lower in the CHM eyes with CSs in both 6 mm and 1500 μm choroidal analysis, although the difference was not significant (all *p* > 0.05, Table 3). In the CHM group with intraretinal cysts, CVI was slightly greater for either 6 mm or 1500 μm evaluation (*p* > 0.05, Table 3).

## 4. Discussion

Intraretinal cysts represent a common ocular complication in patients with inherited retinal diseases including CHM, RP, gyrate atrophy, enhanced S-cone syndrome, and X-linked retinoschisis [18]. The prevalence of cystoid changes in eyes with CHM ranges from 20% to 63% as detected by SD-OCT [5,7].

Various factors and mechanisms have been proposed for the onset of CSs in CHM patients including impairment of the blood–retinal barrier, tangential vitreous traction, and Muller cells degeneration [7]. Although all these mechanisms might work in aggregate, involvement of the choroid in the pathophysiology of the intraretinal fluid has been discussed minimally in the literature.

To our knowledge, this is the first study that investigates the potential involvement of the choroid in the development of intraretinal cysts in patients with CHM. We found no significant differences in terms of subfoveal CT, CVI, and all subcomponents (TCA, SCA, and LCA) between eyes with and without CSs, and this was true for both 6 mm and 1500 μm analyzed choroidal areas. This means that the choroidal vascular structure did not differ between eyes with or without cysts. It is known that in CHM patients, CT is normal in early stages of the disease and tends to decrease with age [19]. Nevertheless, despite being a robust tool in clinical research, CT only reflects the total choroidal vasculature with no distinctions between the two stromal and luminal vascular components. Based on this concept, CVI evaluation is more informative compared to CT measurement alone [9].

The CVI, a ratio of LCA over TCA, has been applied to various chorioretinal diseases [10,20,21], including many inherited retinal dystrophies [12,22]. Wei et al. found a decreased CVI in patients with RP, cone–rod dystrophy, Stargardt disease, Bietti crystalline dystrophym, and Best disease [22]. Recently, Iovino et al. investigated the choroidal features associated with the presence of CME in eyes with RP, reporting an increased CT and decreased CVI in eyes with intraretinal fluid [12]. The authors speculated that the thicker choroid in RP patients with intraretinal fluid may result from locally increased blood flow secondary to intraocular inflammation, leading to the development of CME [12].

In our study, TCA, LCA, and SCA were all decreased in eyes with CSs at 6 mm choroidal analysis, and all these findings were also reflected at 1500 μm choroidal analysis, although none of these differences were significant. Moreover, as CVI is a ratio of LCA over TCA, it only changes significantly if one of the two choroidal parameters varies more than the other. Murro et al. recently reported no differences in the CVI between young CHM patients and controls, proposing a simultaneous, proportional impairment of both the stromal and luminal components of the choroid in the early stage of the disease [11]. Notwithstanding, they did not make any distinctions between eyes with or without CSs.

Regarding the location of the cysts, our results confirmed the observations of previous authors who described INL as the major cystoid location [5,7]. Additionally, some authors reported the presence of degenerative cysts mainly located in the INL at the transition zone where RPE and outer retinal layers are severely damaged with underlying areas of residual choroidal tissue [8]. Injury of the Muller cells was proposed as the main pathogenetic factor leading to CSs onset in CHM patients [8].

The damage of the outer retinal bands (EZ and ELM) under the cysts in 88.2% of the eyes found in our cohort seems to further confirm this hypothesis.

Conversely, cystoid changes in RP and X-linked retinoschisis are usually located over preserved outer retina layers [23,24,25].

ORTs or rosette-like structures have been described in the setting of several chorioretinal disorders [26,27,28]. Although the pathogenic mechanism leading to the formation of the tubules remains uncertain, onset of ORTs correlates with the damage of photoreceptors and/or RPE, and has been proposed as a prognostic feature of disease severity [26]. The higher incidence of ORTs in the CS group, together with the outer retinal band alterations under the cysts, seems to indicate a degenerative nature of the CSs in patients with CHM.

Macular cysts in retinal dystrophy may develop because of tissue loss secondary to disruption of the retinal architecture, and typically these cystoid changes show minimal or no leakage on fluorescein angiography (FA) [29].

In noninflammatory optic neuropathies including dominant optic atrophy and Leber hereditary optic neuropathy, cysts in the INL are thought to be due to transsynaptic degeneration [30]. Moreover, cysts seem to be more prevalent in younger patients with an intact vitreous base, suggesting that vitreous traction may have a role in CSs development [30].

A discrepancy between the presence of cysts on OCT and visual acuity in some patients with retinal dystrophy has also been reported [31]. In fact, as seen in our study, increased CMT does not always correlate with worse BCVA.

Immunochemical studies showed the presence of carbonic anhydrase enzymes in the RPE, as well as in different cells of the neural retina [32,33].

Carbonic anhydrase inhibitors mediate RPE fluid transfer across the outer blood–retinal barrier, and this may further support the choroid as an important therapeutic target in eyes with CME. Several studies confirmed the efficacy of carbonic anhydrase inhibitors in several retinal dystrophies complicated by CSs [18,29,34], although the evidence of these clinical effects on patients with CHM is very limited [18,35]. Progressive atrophy of the RPE and the choroid may explain the limited action of carbonic anhydrase inhibitors in CHM patients.

The main limitations of the current study included its retrospective nature and the relatively small sample size. Subgroup analyses may not have been powered to identify statistically significant differences. Additionally, we excluded eyes with incomplete visualization of the choroid. Patients with advanced-stage CHM often have optic media opacities, and it is possible that excluding such cases may represent an additional potential confounder. FA was not performed to identify any macular leakage, and the presence of a posterior vitreous detachment was not ascertained with OCT or ocular ultrasound.

In conclusion, our study did not show any significant association of various choroidal parameters with the presence of CSs in CHM patients. Our results suggest that intraretinal cysts overlying damaged outer retinal layers may be the result of degenerative more than exudative processes. Further studies are warranted to provide new insights into the intraretinal fluid pathogenesis and to facilitate the development of more efficacious treatment strategies.

## Figures and Tables

**Table 1:** Genetic features of choroideremia patients.

ID	Presence of Cystoid Spaces	CHM Pathogenic Variant (NM_000390.3; NP_000381.1)
P1	YES	c.940 + 1G > T
P2	YES	c.1-?_*3450+?del
P3	YES	c.1520A > G (p.His507Arg)
P4	YES	Deletion encompassing exons 6 and 7
P5	YES	c.1414-?_1510+?del
P6	YES	c.1414-?_1510+?del
P7	YES	c.1166 + 1G > C
P8	YES	c.1166 + 1G > C
P9	YES	c.315_318del (p.Ser105Argfs)
P10	YES	c.580_581ins (p.Asp184Glufs)
P11	YES	c.1029delG (p.Met343llefs)
P12	YES	c.106 + 1G > T (p.Gly17Glufs37*)
P13	YES	c.877C > T (p.Arg293*)
P14	YES	c.877C > T (p.Arg293*)
P15	YES	c.1651delTACTT
P16	YES	c.(?_49 + 1)_(1609 + 1_1610-1)del
P17	YES	c.877C > T (p.Arg293*)
P18	NO	Deletion encompassing exons 6 and 7
P19	NO	Deletion encompassing exons 6 and 7
P20	NO	c.525_526del(p.Glu177Lysfs)
P21	NO	c.580_581ins (p.Asp184Glufs)
P22	NO	c.941-2A > G
P23	NO	c.969T > A (p.Tyr323*)
P24	NO	c.808C > T(p.Arg270*)
P25	NO	c.969T > A (p.Tyr323*)
P26	NO	c.(?_49 + 1)_(1609 + 1_1610-1)del
P27	NO	c.1350-1G > A
P28	NO	c.969T > A (p.Tyr323*)
P29	NO	c.820-2A > G
P30	NO	c.1245-?_1962 + del*(p.Cys416*)
P31	NO	c.31S_318del, p. Ser10Sfs
P32	NO	c.315_318del
P33	NO	c.1029delG (p.Met343llefs)

CHM = choroideremia, ID = identity.

**Table 2:** Patients demographics and comparison of structural retinal and choroidal characteristics between CHM patients with and without CSs.

	CHM Eyes with CSs (*n* = 17)	CHM Eyes without CSs (*n* = 16)	*p*-Value	B–H Adjusted α
Age (Avg ± STD)	37.3 ± 11.6	31.4 ± 16.7	0.25	0.020
BCVA (LogMAR, Avg ± STD)	0.11 ± 0.20	0.20 ± 0.57	0.54	0.043
Central macular thickness (μm, Avg ± STD)	272.3 ± 57.7	232.1 ± 69.3	0.08	0.007
Sub-foveal choroidal thickness (μm, Avg ± STD)	187.9 ± 71.9	215.6 ± 67.9	0.26	0.023
Ellipsoid zone integrity in the foveal region (#, %)	8 (47.1%)	12 (75.0%)	0.16	0.013
External limiting membrane integrity in the foveal region (#, %)	11 (64.7%)	12 (75.0%)	0.71	0.050
Tubulation (#, %)	17 (100%)	11 (68.7%)	0.02	0.003

Data are expressed as mean ± standard deviation or counts (frequency) for continuous and categorical variables, respectively. CHM = choroideremia, CSs = cystoid spaces, Avg = Average, STD = Standard deviation, BCVA = best corrected visual acuity.

**Table 3:** Choroidal parameters in CHM patients with and without CSs in the total 6 mm scan and central 1500 μm under the fovea.

Choroidal Parameters for the Total 6 mm Scan	CHM Eyes With CSs (*n* = 17)	CHM Eyes Without CSs (*n* = 16)	*p*-Value	B–H Adjusted α
Choroidal Vascularity Index (%, Avg ± STD)	68.1 ± 4.4	66.4 ± 4.0	0.28	0.034
Total Choroidal Area for (mm^2^, Avg ± STD)	0.89 ± 0.43	1.09 ± 0.59	0.28	0.030
Luminal Choroidal Area (mm^2^, Avg ± STD)	0.61 ± 0.29	0.71 ± 0.38	0.36	0.037
Stromal Choroidal Area (mm^2^, Avg ± STD)	0.29 ± 0.15	0.37 ± 0.22	0.21	0.017
**Choroidal Parameters for the 1500 μm under the fovea**				
Choroidal Vascularity Index (%, Avg ± STD)	70.4 ± 6.1	67.4 ± 5.0	0.14	0.010
Total Choroidal Area for (mm^2^, Avg ± STD)	0.28 ± 0.13	0.31 ± 0.15	0.61	0.047
Luminal Choroidal Area (mm^2^, Avg ± STD)	0.18 ± 0.09	0.2 ± 0.1	0.52	0.040
Stromal Choroidal Area (mm^2^, Avg ± STD)	0.08 ± 0.05	0.1 ± 0.06	0.26	0.024

Data are expressed as mean ± standard deviation. CHM = choroideremia, CSs = cystoid spaces, Avg = average, STD = standard deviation.

## Data Availability

Data available from authors.
